# Joint developmental trajectories of depressive symptoms, perceived stress in adolescence and their relationship with smartphone addiction

**DOI:** 10.3389/fpsyt.2025.1573261

**Published:** 2025-07-01

**Authors:** Cai Zhang, Zhiqian Zhang, Hanning Lei, Xia Wang, Yun Wang

**Affiliations:** ^1^ Collaborative Innovation Center of Assessment for Basic Education Quality, Beijing Normal University, Beijing, China; ^2^ Haidian Institute of Education Sciences, Beijing, China; ^3^ Faculty of Psychology, Beijing Normal University, Beijing, China; ^4^ State Key Laboratory of Cognitive Neuroscience and Learning, Beijing Normal University, Beijing, China

**Keywords:** depressive symptoms, perceived family stress, perceived academic stress, joint developmental trajectories, smartphone addiction

## Abstract

**Introduction:**

Mental health issues frequently cluster during adolescence, necessitating a deeper understanding of their developmental trajectories and their impact on behavioral outcomes like smartphone addiction. Based on diathesis-stress model and I-PACE model, this study examined the joint trajectories of depressive symptoms, family stress perception, and academic stress perception among adolescents and their relationship with smartphone addiction.

**Methods:**

12,074 Chinese adolescents (47.9% girls; *M*_age_ = 12.28 ± 0.45 years) were assessed for depressive symptoms, perceived family stress and academic stress from seventh to ninth grade, with smartphone addiction measured in the final year.

**Results:**

Using a group-based multi-trajectory model, five distinct trajectories were identified: Low-Stable (23.8%), Moderate-Stable (45.9%), Moderate-Increasing (14.8%), High-Decreasing (11.2%), and High-Stable (4.3%). Gender differences were evident, as girls were more likely than boys to belong to higher stress or depressive symptoms trajectories compared to the low-stable group. Smartphone addiction scores differed significantly across trajectory groups, ranging from high-stable to low-stable. Girls consistently showed higher smartphone addiction scores, although the interaction between gender and trajectory group was not significant.

**Conclusion:**

These findings highlight the importance of recognizing both shared and individual patterns of adolescent mental health development. Schools and families should implement tailored interventions to address depression and stress, particularly for vulnerable groups such as girls, to mitigate the risk of smartphone addiction and promote overall mental well-being.

## 1 Introduction

Adolescent mental health has garnered increasing global attention, with depressive symptoms and perceived stress emerging as two interrelated yet distinct psychological challenges. Between 2011 and 2020, the global prevalence of depressive symptoms among adolescents reached 37% (1), while 25% of individuals aged 10 to 19 experienced significant stress-related distress (2, 3). These mental health concerns profoundly impact adolescent development, increasing risks of substance abuse, academic underachievement, and suicidal tendencies (4). Although numerous studies have demonstrated a strong correlation and high comorbidity between depressive symptoms and perceived stress (5, 6), but significant gaps remain in understanding their developmental trajectories.

Adolescence, as a critical period of psychological development, is characterized by hormonal changes, physical maturation, and cognitive development (7). These changes may lead to fluctuations in mental health problems like depressive symptoms and perceived stress, which may follow distinct trajectories (8). These different trajectory patterns may reflect underlying etiological differences and lead to varying developmental outcomes, playing a crucial role in understanding the significance of depression and perceived stress in adolescent development (9). However, few studies have examined the joint developmental patterns of these two constructs over time or the heterogeneity that may exist among individuals, particularly in early adolescence—a critical period for mental health development. This is especially significant within China’s unique educational context, where junior high school students face a surging academic burden, accompanied by a sharp increase in academic pressure, family stress, and depressive symptoms.

The present study addresses these gaps by investigating the relationship between mental health trajectories and emerging behavioral challenges, particularly smartphone addiction. According to a meta-analysis of 24 countries, smartphone addiction was increasing across adolescents and young adults over the last decade, especially in China (10). Research demonstrates its association with various negative outcomes, including lower academic performance (11), interpersonal conflicts (12), and impaired self-regulation (13). Both depressive symptoms and perceived stress are highly associated with smartphone addiction (14–16). Building on developmental psychopathology framework, this study adopts a person-centered approach to examine the joint developmental trajectories of depressive symptoms and perceived stress among Chinese 7th graders and their relationship with smartphone addiction. The findings will provide valuable insights for developing targeted interventions and advancing our understanding of adolescent mental health development.

### 1.1 Independent development trajectories of depressive symptoms and perceived stress

Depressive symptoms refer to a condition that individuals exhibit certain depressive characteristics without meeting the diagnostic criteria for depression. The symptoms primarily include a persistent low mood and loss of interest, often accompanied by physical symptoms (e.g., sleep disturbances, abnormal appetite) and suicidal ideation (17). Due to the acceleration of physiological development and changes in hormone levels, adolescents’ depressive symptoms often follow heterogeneous developmental trajectories, reflecting variations in onset, persistence, and change over time (18, 19). Empirical research has consistently identified several distinct trajectory patterns in adolescent depressive symptoms. Musliner et al.’s comprehensive review of 25 trajectory studies revealed three primary stable patterns: a low-symptom trajectory (typically representing over 45% of samples), a high-symptom trajectory (generally comprising less than 7% of samples), and a moderate-symptom trajectory (ranging from 16% to 61% across studies) (8). Additionally, dynamic trajectories characterized by symptom fluctuation have been well-documented. For instance, Brendgen et al. identified four developmental patterns in North American adolescents aged 11-14, ranging from stable low/moderate symptoms to clinically significant patterns showing either early adolescent onset or progressive worsening from childhood (20). Similarly, Tang et al. revealed four distinct trajectories in a longitudinal study of Chinese adolescents (aged 16-22) over five years, including stable low, moderate-high, persistent high, and increasing symptom patterns (21). These findings have been consistently replicated across various cultural contexts (22, 23). Building on the above findings, we propose that the development of depression in adolescents primarily includes trajectories of consistently low, moderate, and high depressive symptoms, as well as developmental processes characterized by an increase from lower to higher symptoms or a sustained decrease from higher symptoms.

Perceived stress is defined as an individual’s subjective evaluation of the significance of stressors or life events (24). Perceived stress is a critical factor influencing adolescent development and follows diverse trajectories, often shaped by developmental transitions and external pressures. For many adolescents, stress remains at manageable levels, but for others, it intensifies significantly during critical periods, such as the transition to college (25). Currently, most studies focus primarily on the short-term or long-term effects of stress on adolescents, with limited research examining the developmental trajectories of stress perception. One exception is the study by Schmeelk-Cone and Zimmerman, which investigated 421 African American adolescents for 5 years and identified four distinct stress trajectory patterns: consistently low stress (30%), consistently high stress (8%), and trajectories characterized by an increase (33%) or decrease (29%) in stress perception after starting at moderate levels (26).

In addition, as essential environments for adolescent development and social interaction, family and school significantly shape adolescents’ lives, with stressors from these different contexts exerting distinct impacts (27, 28). Family stress primarily stems from conflicts between adolescents and family members or their experiences of parental discipline and demands, whereas academic stress mainly often arises from students’ concerns about their inability to meet expected academic standards, leading to psychological distress (29–31). Previous studies have shown that family stress is significantly associated with adolescents’ internalizing problems (e.g., anxiety and depression) and may restrict their autonomy through family conflicts and parental control, leading to emotional distress and low self-esteem. In contrast, academic stress not only contributes to internalizing problems but also affects students’ social adaptation and externalizing problems (32, 33). Given the differences in the stressors, impact pathways, and temporal dynamics of family and academic stress, examining their developmental trajectories separately allows for a deeper understanding of how stress influences adolescents in distinct ways. This, in turn, provides a scientific basis for more targeted psychological support and educational policies, which are crucial for developing effective interventions to address the unique stressors in different domains.

### 1.2 Joint developmental trajectories of depressive symptoms and perceived stress

A substantial body of empirical research has demonstrated the bidirectional relationship between adolescent depression and perceived stress (5, 6), indicating that higher levels of stress can lead to increased depressive symptoms, while elevated depressive symptoms may also contribute to a greater occurrence of stressful life events, especially in adolescents (34–36). Studies have shown that individuals with depression experience 2.5 times more stressful life events compared to non-depressed individuals, with 80% of depression cases being triggered by stressful life events (36, 37). Therefore, integrating the joint trajectory of perceived stress and depression provides a more comprehensive understanding of their dynamic relationship. Further exploration of the joint developmental trajectories of perceived stress and depression can facilitate the identification and monitoring of shared developmental characteristics across multiple factors, which hold significant clinical implications (38).

From the perspective of developmental trajectories, mental health problems such as depressive and anxious symptoms often exhibit similar co-occurring patterns and share comparable developmental trajectories. For example, a study explored the joint developmental trajectories of early childhood shyness, anxiety, and depression. The results indicated a high degree of overlap between the developmental trajectories of depression and anxiety, with 39.8% of individuals displaying persistently low levels of depression and anxiety throughout childhood and early adolescence, while 5% exhibited persistently high levels of both anxiety and depression (39). Although research on the joint developmental trajectories of depressive symptoms and perceived stress remains limited, considering their interrelationship and the similarities in developmental patterns of mental health problems, they may exhibit parallel trajectories when examined together. Specifically, based on findings from empirical studies on independent developmental trajectories of depressive symptoms and perceived stress, the patterns may be characterized by consistently low and high levels, increases from low levels, and decreases from high levels (20, 21, 26).

According to the diathesis-stress model, depressive symptoms and perceived stress may develop synergistically through the dynamic interaction between individual vulnerabilities and environmental stressors (40). Individuals with limited exposure to stressors or stronger emotional regulation capacities tend to maintain consistently low levels of both depressive symptoms and perceived stress over time, forming a “low depression–low stress–stable” developmental trajectory. In contrast, those with cognitive vulnerabilities or difficulties in emotion regulation may become increasingly sensitized to stress under repeated exposure to high-intensity stressors, resulting in escalating depressive symptoms and heightened stress perception. This can result in a “depression–stress increasing” trajectory (41, 42). Moreover, some individuals may experience concurrent reductions in depressive symptoms and perceived stress following positive lifestyle adjustments or enhanced social support, which help reduce stress exposure and strengthen coping resources, ultimately leading to a “depression–stress–decreasing” developmental pathway.

Moreover, numerous studies have shown that there are gender differences in the developmental trajectories of depression and stress, girls are more susceptible to experiencing depressive emotions and higher levels of stress compared to boys (43, 44). One study examining the developmental trajectories of depression among adolescents aged 7 to 12 of different genders found that girls consistently exhibited significantly higher depression scores than boys each year. Furthermore, this depressive state was associated with stress, as changes in stressful life events accounted for the variations in depressive symptoms (45). Thus, it can be inferred that girls are more likely to perceive stress from negative life events, which in turn has a greater impact on their mental health. As a result, their joint trajectory development is more likely to exhibit patterns of consistently high levels or increasing trends.

### 1.3 The relationship between the joint developmental trajectories of depressive symptoms, perceived stress, and smartphone addiction

A comprehensive framework that can effectively explain the relationship between depression, stress perception, and smartphone addiction is the interaction of person-affect-cognition-execution (I-PACE) model (46, 47). According to the model, predisposing factors (e.g., psychopathological features) are relatively stable internal characteristics that increase an individual’s vulnerability to developing specific internet use disorders. Among these factors, depressive symptoms and perceived stress have been recognized as particularly important contributors to the development of smartphone addiction. Individuals who experiencing depressive symptoms and heightened stress levels are more likely to adopt impulsive coping strategies, often turning to smartphones as a tool for regulating negative emotions. This tendency can increase their dependence on smartphones and, over time, lead to addiction (46). Cross-sectional studies support the I-PACE model by highlighting a positive association between depression, stress, and smartphone addiction, adolescents who report higher levels of depression and stress perception are more prone to excessive smartphone use (11, 48, 49).

In addition, from a longitudinal perspective, individuals’ depressive symptoms and perceived stress may fluctuate over time, and varying patterns of these changes may produce inconsistent effects on smartphone addiction. This phenomenon can be explained by equifinality and multifinality within the developmental psychopathology framework. Equifinality emphasizes that different trajectory can lead to same outcomes through different mechanisms while multifinality indicates that same starting point can lead to different outcomes under different conditions, such as distinct personality traits or generational contexts (50, 51). For example, Martinez et al. found that adolescents with different depressive symptom trajectories were at different risks of alcohol use disorder. Consistently higher levels of depressive symptoms were at significantly greater risk of alcohol use disorder relative to other trajectories. Additionally, those whose depressive symptoms started low but increased over time were at a slightly higher risk of alcohol use disorder than those with persistently low depression levels (52). This perspective underscores the necessity of identifying distinct trajectory types of depressive symptoms and perceived stress and examining whether these differentiated patterns are associated with varying levels of smartphone addiction.

Traditional empirical research has predominantly adopted a variable-centered approach, which focuses on associations between variables at the group level. For instance, prior longitudinal studies revealed that adolescents with sustained high levels of depression are more likely to exhibit increasing levels of smartphone addiction (53). However, this perspective always leads to ecological fallacy, where conclusions drawn at the group level are directly applied to individuals, ignoring the heterogeneity among individuals. To address these limitations, recent studies have adopted a person-centered approach, such as latent trajectory analysis, to explore how developmental trajectories of different adolescent characteristics influence smartphone addiction. For example, a three-year longitudinal study on Spanish adolescents examined the impact of changes in social support on smartphone addiction. By establishing a latent growth model, the study found that an increasing trajectory of social support was associated with a reduction in smartphone addiction (54). However, no research has investigated the joint developmental trajectories of depression, stress, and other internalizing problems in relation to smartphone addiction, or whether distinct trajectories are associated with different levels of smartphone addiction. This study seeks to fill this gap.

### 1.4 Current study

Building on established theoretical frameworks in developmental psychopathology, this study employs group-based multi-trajectory modeling to systematically examine the joint developmental patterns of depressive symptoms and two interrelated yet distinct domains of adolescent stress: family-related and academic-related stressors. As the first investigation to integrate these three constructs within a person-centered analytical framework, our research further explores how distinct trajectory subgroups differentially correlated with risks of smartphone addiction—a prevalent externalizing behavior with escalating public health concerns. The findings aim to provide novel empirical evidence for designing developmentally sensitive prevention strategies targeting both mental health promotion and digital behavior regulation in adolescents.

## 2 Materials and methods

### 2.1 Sample & procedure

The study utilized longitudinal data from a district-level adolescent mental health assessment initiative commissioned by the Beijing Municipal Education Commission. The data were collected in Beijing, China. Employing school-based sampling, all seventh-grade students within the district were recruited through their respective middle schools. Data collection occurred via a secure online platform, with individualized access credentials distributed to participants. Following written parental consent, assessments were administered during regular school hours under teacher supervision in school computer laboratories. Participants received standardized instructions emphasizing data confidentiality and voluntary participation. The research protocol received ethical approval from the Research Ethics Committee of the Collaborative Innovation Center of Assessment for Basic Education Quality at Beijing Normal University (Approval No: 2022-58).

Three-wave panel data were collected between November 2020 and November 2022, with assessment waves spaced at 12-month intervals (Wave 1: Nov 2020; Wave 2: Nov 2021; Wave 3: Nov 2022). The comprehensive assessment battery included validated psychological scales administered through standardized questionnaires. From the initial cohort of 21,186 seventh-graders, longitudinal retention rates were 83.2% (*N* = 17,636) at Wave 2 and 68.5% (*N* = 12,074) at Wave 3, yielding an overall retention rate of 57.0% across three years. Attrition analysis comparing retained (*N* = 12,074) and attrited (*N* = 9,112) participants revealed no significant differences in gender distribution (χ² = 1.40, *p* = 0.236) or family socioeconomic status (*t* = 0.55, *p* = 0.584). However, compared to those who remained in the study, participants who dropped out of the study reported significantly higher baseline depressive symptoms (*t* = -6.64, *p* < 0.001) and higher baseline perceived stress, including academic stress (*t* = -6.44, *p* < 0.001) and family stress (*t* = -6.08, *p* < 0.001). The final analytical sample comprised 12074 adolescents (47.9% girls; *M*_age_ = 12.28 years, SD = 0.45 at baseline) distributed across 67 public middle schools. Students’ self-perceived family economic status is at a moderate level.

### 2.2 Measures

#### 2.2.1 Depressive symptoms

Depressive symptoms were assessed using Patient Health Questionnaire (PHQ-9) (55). This scale consists of 9 items as seeing how many times in the past 2 weeks they felt have experienced issues such as feeling down, having trouble sleeping, or difficulty concentrating. For example, “feeling unmotivated or uninterested in activities” “Having difficulty falling asleep, experiencing restless sleep, or sleeping excessively”. The items were scored on 4-point scale, from 0 “not at all” to 3 “nearly every day”. The total depression score is obtained by summing the scores of all items for each student, ranging from 0 to 27, higher scores indicate more severe depressive symptoms. Cronbach’s alpha for our sample ranged from 0.898~0.903.

#### 2.2.2 Perceived stress

The Adolescent Life Events Scale was used to measure family and academic stress perception (56). The original scale consisted of 27 items. Based on the project requirements and objectives, we selected 7 items that specifically reflect family stress and academic stress, which measured the occurrence and importance of family and academic stress events over the past year, such as financial difficulties, family conflicts, academic pressure, and exam failure. For example, “My parents have overly high expectations of me” “Failing an exam or performing poorly in an exam”. All items are scored on 5 degrees, from 1 “no impact” to 5 “extreme impact”. The average scores for family stress perception and academic stress perception were obtained by calculating the mean of each student’s scores on the respective items, ranging from 1 to 5, higher scores indicate a greater perception of stress. Cronbach’s alpha for our sample ranged from 0.687~0.716 (perceived family stress), 0.818~0.823 (perceived academic stress).

#### 2.2.3 Smartphone addiction

Smartphone addiction was measured using the 15 items Smartphone addiction scale (57). The scale assessed the participants’ mobile phone addiction across four dimensions: interference with daily life, tendency toward virtual life, withdrawal response, and tolerance level, rated from 1 “very disagree” to 4 “very agree”. The items were like “I feel nervous and uneasy when my phone or other electronic devices are not around me” “I can control the amount of time I spend using my phone and other electronic devices.” The total score for smartphone addiction is obtained by summing all item scores, ranging from 15 to 60, higher scores indicate a greater level of smartphone addiction. Cronbach’s alpha for our sample was 0.814.

### 2.3 Data analytic strategy

First, a correlation analysis was conducted to examine the relationships among depressive symptoms, perceived family stress, perceived academic stress, and smartphone addiction. Second, the independent developmental trajectories of depressive symptoms, perceived family stress, and perceived academic stress were determined using latent class growth model (LCGM) in Mplus. Next, a group-based multi-trajectory model with covariates was constructed in STATA to analyze the joint developmental trajectories of depression, family stress perception, and academic stress perception, as well as the role of gender (38). Group-based multi-trajectory model is an extension of group-based trajectory modeling (GBTM). It divides multi-variable longitudinal data into several latent groups using a finite mixture model, where each group is defined by the joint trajectories of multiple indicators. Finally, multivariate analysis of variance (ANOVA) and multiple group comparisons were conducted to examine differences in smartphone addiction scores across trajectory groups and assess the impact of gender. Multivariate ANOVA and multiple group comparisons were conducted to examine differences in smartphone addiction scores across different trajectory groups and assess the impact of gender.

## 3 Results

### 3.1 Descriptive statistics

**Table 1** presents the descriptive statistics and intercorrelations among study variables across three waves. Cross-temporal analyses revealed consistent positive associations between depressive symptoms, family-related stress, and academic-related stress, with all pairwise correlations reaching statistical significance (*r* range = 0.23-0.61, all *p* <.001). These psychosocial factors demonstrated stable positive correlations with smartphone addiction throughout adolescence (*r* range = 0.18-0.47, all *p* <.001). Notably, gender differences emerged as a systematic pattern across measurement waves. Girls showed elevated scores in depressive symptoms (*r* = 0.07-0.09), academic stress perception (r = 0.04-0.05), and smartphone addiction severity (*r* = 0.05).

**Table 1:** Descriptive statistics and bivariate correlations between key variables.

	1	2	3	4	5	6	7	8	9	10
1.Depressive symptoms T1	—									
2.Depressive symptoms T2	0.48^***^	—								
3.Depressive symptoms T3	0.39^***^	0.51^***^	—							
4.Perceived family stress T1	0.51^***^	0.32^***^	0.26^***^	—						
5.Perceived family stress T2	0.33^***^	0.50^***^	0.34^***^	0.41^***^	—					
6.Perceived family stress T3	0.26^***^	0.34^***^	0.49^***^	0.33^***^	0.45^***^	—				
7.Perceived academic stress T1	0.47^***^	0.30^***^	0.25^***^	0.55^***^	0.32^***^	0.26^***^	—			
8.Perceived academic stress T2	0.29^***^	0.46^***^	0.32^***^	0.29^***^	0.60^***^	0.33^***^	0.44^***^	—		
9.Perceived academic stress T3	0.23^***^	0.32^***^	0.48^***^	0.24^***^	0.34^***^	0.61^***^	0.35^***^	0.47^***^	—	
10.Smartphone addiction	0.26^***^	0.35^***^	0.47^***^	0.22^***^	0.27^***^	0.39^***^	0.18^***^	0.22^***^	0.37^***^	—
Sex (1 = girl)	0.07^***^	0.09^***^	0.09^***^	0.03^**^	0.02^*^	0.01	0.04^***^	0.05^***^	0.04^***^	0.05^***^
M (scores)	4.88	5.33	5.58	0.76	0.88	0.80	1.93	1.82	1.81	30.12
SD	5.57	5.58	5.60	0.10	1.05	1.03	1.18	1.15	1.15	8.58

*N* = 12074, T1 is Time 1, T2 is Time 2, T3 is Time 3. *p < 0.01, **p < 0.01, and ***p < 0.001, and the same below.

### 3.2 Joint developmental trajectories of depressive symptoms and perceived stress

We first established separate trajectory models for depressive symptoms, perceived family stress, and perceived academic stress, comparing models with 2 to 5 latent classes. Based on previous relevant studies, the final model selection was determined by Log-likelihood, AIC (Akaike Information Criterion), BIC (Bayesian Information Criterion), aBIC (Adjusted Bayesian Information Criterion), and entropy. The final trajectory modeling results for each variable are shown in **Table 2**. We compared models with different trajectory classifications. Taking depressive symptoms as an example, we observed that the change in AIC and BIC was the largest when moving from a three-class to a four-class model, whereas the LRT for the five-class model was not significant (*p* = 0.323). Additionally, the posterior probability (which requires the average posterior probability of group members to be > 0.7) and the minimum subgroup proportion (each subgroup must account for no less than 5%) of the four-class model met the required criteria, leading to its selection as the final model (58). The same criteria were applied to perceived family stress and perceived academic stress.

**Table 2:** Model indices for the latent class growth models of depressive symptoms, perceived family stress and perceived academic stress.

Model	Log-likelihood	AIC	BIC	aBIC	Entropy	LRT	bLRT
Depressive Symptoms							
2	-109201.69	218423.37	218497.36	218465.58	0.884	<0.001	<0.001
3	-108069.55	216167.09	216270.68	216226.18	0.841	<0.001	<0.001
4	-107152.84	214341.68	214474.85	214417.65	0.875	0.005	<0.001
5	-106329.27	212702.54	212865.32	212795.40	0.881	0.323	<0.001
6	-105650.35	211352.71	211545.08	211462.45	0.864	0.204	<0.001
Perceived Family Stress							
2	-48130.65	96281.30	96355.29	96323.51	0.882	<0.001	<0.001
3	-46829.32	93686.64	93790.22	93745.73	0.896	<0.001	<0.001
4	-45539.82	91115.64	91248.82	91191.62	0.902	<0.001	<0.001
5	-45539.82	91123.64	91286.42	91216.50	0.916	<0.001	<0.001
6	-44821.66	89695.31	89887.68	89805.05	0.90	0.023	<0.001
Perceived Academic Stress							
2	-54066.62	108153.25	108227.23	108195.45	0.671	<0.001	<0.001
3	-53482.73	106993.46	107097.04	107052.55	0.716	<0.001	<0.001
4	-53292.23	106620.46	106753.64	106696.44	0.747	<0.001	<0.001
5	-53310.16	106664.32	106827.10	106757.184	0.798	<0.001	<0.001
6	-53090.636	106233.273	106425.642	106343.017	0.804	<0.001	<0.001

*AIC*, Akaike Information Criterion; *BIC*, Bayesian Information Criterion; *aBIC*, adjusted Bayesian Information Criterion; *LRT*, Likelihood Ratio Test; *bLRT*, bootstrap Likelihood Ratio Test; and the same below.

Based on the independent trajectory grouping of depressive symptoms, perceived family stress, and academic stress, we determined that at least 4 distinct joint developmental trajectories of depressive symptoms, perceived family and academic stress were required. We compared the joint trajectory groups, as shown in **Table 3**. Based on the average posterior probability and the minimum subgroup proportion, we observed that the change in AIC and BIC values was the largest from the four-class to the five-class model. Additionally, in the six-class model, one group accounted for only 1.5% of the total sample. Therefore, we ultimately selected the five-class model as the classification for the joint developmental trajectories of depressive symptoms, perceived family stress, and perceived academic stress, named after the initial state and developmental trajectories over the 3 years.

**Table 3:** Model indices for the joint developmental trajectory modeling of depressive symptoms, perceived family stress and perceived academic stress.

Group	Log-likelihood	AIC	BIC	aBIC	Entropy
2	-200655.70	-200678.70	-200763.79	-200789.06	0.807
3	-197701.01	-197735.01	-197860.79	-197898.14	0.788
4	-196792.01	-196837.01	-197003.48	-197052.92	0.764
5	-195780.73	-195836.73	-196043.90	-196105.42	0.764
6	-195133.73	-195200.73	-195448.59	-195522.20	0.789

**Figure 1:**
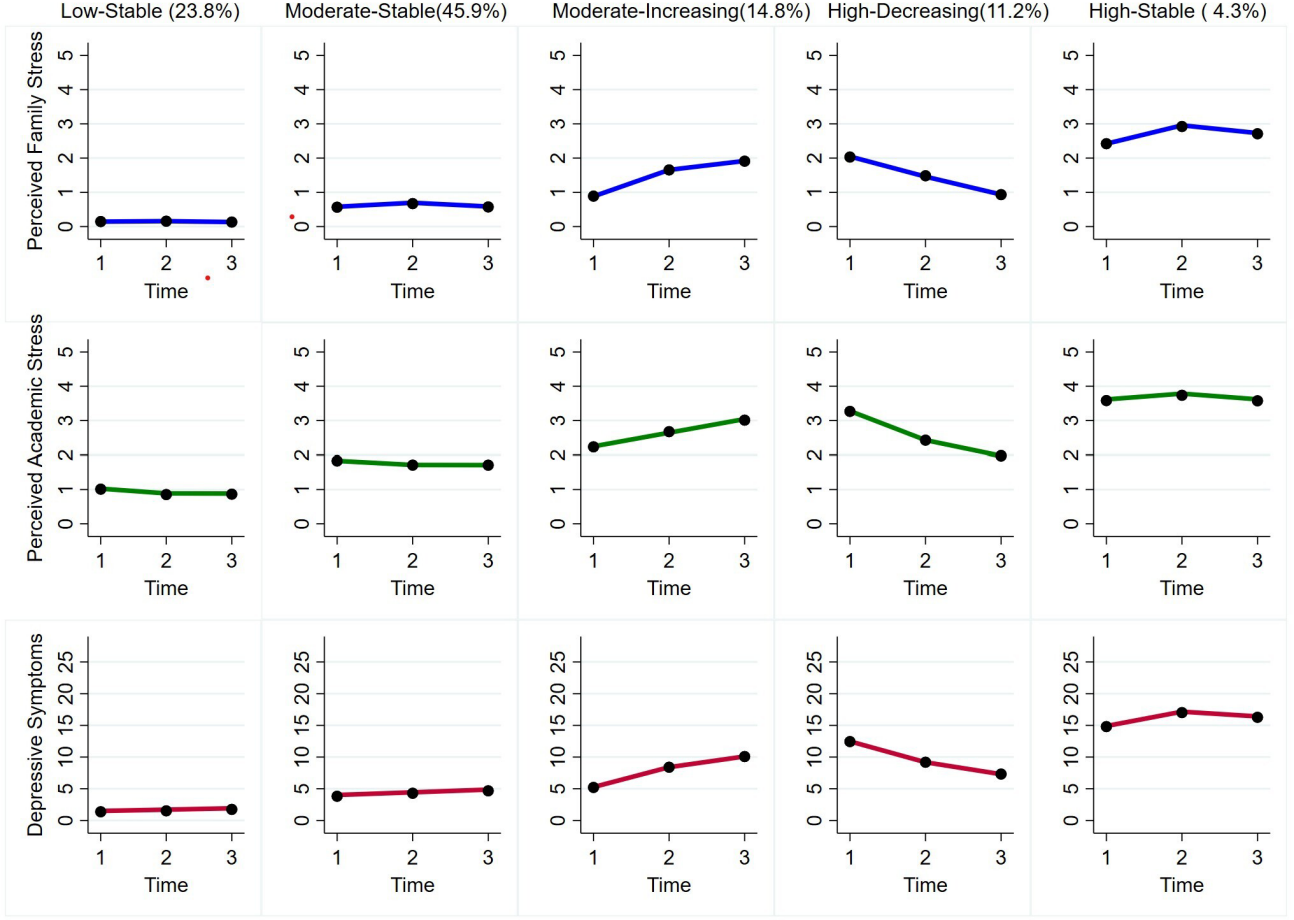
Joint developmental trajectories of depressive symptoms, perceived family stress and perceived academic stress.

(1) Low-Stable Group. This group maintained persistently low levels of depression, family and academic stress throughout junior school, accounting for 23.8% (n = 2825) of the sample, which represents a stable and mentally healthy subgroup without significant emotional problems (2). Moderate-Stable Group. As the largest group, comprising 45.9% (n = 5542) of participants, characterized by consistently moderate levels of the three variables over time. It reflects a population experiencing a certain degree of emotional distress in a relatively stable pattern (3). Moderate-Increasing group. This group started with moderate levels of depression and stress, followed by a steadily increasing trend across the three years, covering 14.8% (n = 1787) of the sample. This group represents adolescents who were experiencing persistent psychological challenges, with symptoms worsening over time for certain reasons, indicating the need for targeted intervention (4). High-Decreasing group. Representing 11.2% (n = 1352) of the participants, this group exhibited high initial levels of depression, family, and academic stress, which gradually declined over the study period. It suggests that these adolescents initially faced significant mental health problems but managed to effectively regulate their emotions and reduce their stress perception in the subsequent years (5). High-Stable group. Accounting for 4.3% (n = 519) of the sample, characterized by persistently high levels of depression and stress across three years. Despite its small size, this group warrants particular attention, as its members consistently experienced substantial emotional distress throughout junior school. The specific developmental trajectories and group classifications are shown in **Figure 1**. Across these trajectories, the developmental patterns of the three variables (depressive symptoms, family stress, academic stress) were generally consistent, indicating that individuals within each group exhibited similar trends in these variables over time.

Moreover, we observed significant gender differences in the occurrence rates of different joint developmental trajectory groups. Therefore, we further analyzed the role of gender using logistic regression. The results indicated that, compared to the Low-Stable group, girls were more likely to belong to Moderate-Stable (*OR* = 1.69, 95%CI = [1.54, 1.85]), Moderate-Increasing (*OR* = 1.77, 95%CI = [1.57, 2.00]), High-Decreasing (*OR* = 1.90, 95%CI = [1.66, 2.17]), and High-Stable (*OR* = 1.88, 95%CI = [1.56, 2.27]) groups. This suggests that, compared to boys, girls may experience more pronounced or persistent challenges related to depressive symptoms, perceived family stress, and perceived academic stress.

### 3.3 Trajectory group and smartphone addiction

**Figure 2:**
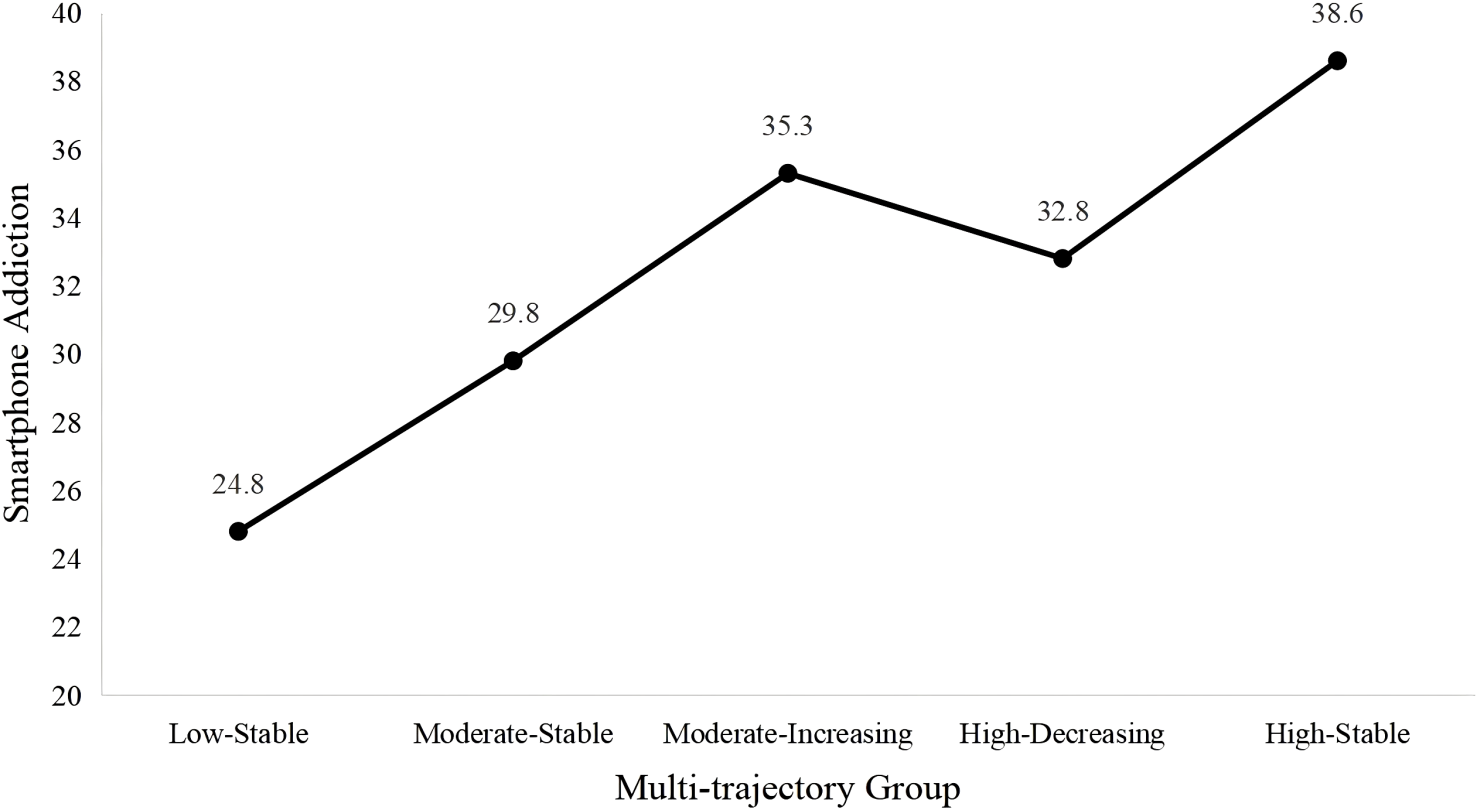
Scores of smartphone addiction across different joint developmental trajectory groups.

We also found significant differences in smartphone addiction scores among adolescents across different joint developmental trajectory groups (F = 730.748, *p* < 0.001), as shown in **Figure 2**. The levels of smartphone addiction, from highest to lowest, were as follows: High-Stable group (*M* = 38.633, SD = 8.714), Moderate-Increasing group (*M* = 35.341, SD = 7.961), High-Decreasing group (*M* = 32.764, SD = 8.363), Moderate-Stable group (*M* = 29.816, SD = 7.773), Low-Stable group *(M* = 24.764, SD = 6.820). The differences between each group are all significant (*p* < 0.001), see **Table 4**. These findings indicate that individuals in the High-Stable group, who consistently experienced high levels of depression, family, and academic pressure, exhibited the highest levels of smartphone addiction. Conversely, those in the Low-Stable group showed the lowest smartphone addiction scores. Additionally, girls had significantly higher smartphone addiction scores compared to boys, regardless of their joint trajectories group. However, the interaction between gender and joint trajectories group membership was not significant (*p* = 0.481), suggesting that the relationship between trajectory group and smartphone addiction scores was consistent across genders.

**Table 4:** Differences in smartphone addiction scores across joint developmental trajectory groups.

Group	MD	SE	p	95%*Cl*
Low Stable vs Moderate Stable	-5.05	0.18	< 0.001	[-5.40, -4.70]
Low Stable vs Moderate Increasing	-10.58	0.24	< 0.001	[-11.04. -10.11]
Low Stable vs High Decreasing	-8.00	0.26	< 0.001	[-8.51, -7.50]
Low Stable vs High Stable	-13.87	0.37	< 0.001	[-14.60, -13.15]
Moderate Stable vs Moderate Increasing	-5.53	0.21	< 0.001	[-5.94, -5.11]
Moderate Stable vs High Decreasing	-2.95	0.24	< 0.001	[-3.41, -2.48]
Moderate Stable vs High Stable	-8.82	0.35	< 0.001	[-9.51, -8.12]
Moderate Increasing vs High Decreasing	2.58	0.28	< 0.001	[2.02, 3.13]
Moderate Increasing vs High Stable	-3.29	0.39	< 0.001	[-4.05, -2.53]
High Decreasing vs High Stable	-5.97	0.40	< 0.001	[-6.65, -5.09]

## 4 Discussion

Our three-year longitudinal study revealed five co-developmental patterns of depressive symptoms, family stress, and academic stress among adolescents: Low-Stable, Moderate-Stable, Moderate-Increasing, High-Decreasing to High-Stable groups, demonstrated consistent patterns of co-development among the three variables, with significant variations in prevalence across the population. Furthermore, smartphone addiction differed significantly across trajectory groups, with the High-Stable group exhibiting the highest addiction levels and the Low-Stable group the lowest. Although girls consistently reported higher smartphone addiction scores, the relationship between trajectory group and addiction levels remained stable across genders. These findings underscore the intertwined development of emotional and perceived stress and their implications for smartphone addiction. Also, the results revealed the following key findings that warrant further discussion.

### 4.1 Characteristics of joint development trajectories of depressive symptoms and perceived stress

The observed developmental trajectories largely align with previous research findings and can be comprehensively explained through the cognitive vulnerability-stress model and the stress adaptation model (59, 60). According to the cognitive vulnerability-stress framework, heightened perceived stress increases the risk of depression by eroding positive experiences and amplifying negative emotional states (11, 61). In turn, depressive symptoms reduce psychological resilience and heighten stress sensitivity, forming a bidirectional reinforcement loop (41). As a result, adolescents experiencing prolonged high levels of stress or depression may become trapped in a vicious cycle, leading to a trajectory characterized by persistently high levels of both stress and depression. Conversely, those with consistently low perceived stress and minimal depressive symptoms are more likely to maintain a stable trajectory with low levels of both variables. Meanwhile, the stress adaptation model suggests that individuals exposed to prolonged stress may gradually adjust their coping strategies, enhancing their emotional regulation skills and psychological resilience (5, 62). This adaptation process can lead to a reduction in depressive symptoms, resulting in a declining trajectory over time. However, for individuals with weaker adaptive capacities, the increasing burden of family and academic stress, combined with limited coping resources, may hinder their ability to effectively manage stress. This accumulation effect exacerbates their depression, leading to a progressively worsening trajectory of stress and depression.

The only difference from previous studies is that the upward trajectory we fitted includes only those individuals transitioning from moderate to higher levels, and no trajectory from low to high levels was observed (21). According to the stress generation framework, the initial severity of depressive symptoms may be positively correlated with subsequent stress perception (63, 64). This may explain why individuals with moderate initial levels of depression and stress perception are more likely to show a significant upward trend, with both depression and stress perception gradually increasing, highlighting the need to focus on this group. Furthermore, unlike the two developmental trajectories of consistently low and high levels of perceived stress and depression commonly identified in previous studies, this study also identifies a trajectory with consistently moderate levels, suggesting substantial variation in stress and depression among different populations within this study’s sample.

In addition, from the distribution of individuals across different trajectories, approximately 80% of the participants maintain their stress and depression trajectories within a manageable range or show a continuous decline, indicating that the mental health of most adolescents is relatively stable or improving. However, around 20% of individuals exhibit trajectories where stress and depressive symptoms remain at higher levels or continue to rise, suggesting that this group experiences higher stress and more severe depression, potentially requiring additional intervention and support. Meanwhile, about 70% of participants show a stable developmental trajectory, indicating that, during this stage, adolescents’ stress and depression levels remain relatively stable without significant fluctuations. It is noteworthy that individuals with consistently moderate levels of stress and depression make up the largest group, which also reflects the characteristics of adolescence. During this stage, adolescents are particularly sensitive both physically and psychologically, facing various changes in their internal and external environments, making them more prone to prolonged adaptation issues. Therefore, researchers should pay particular attention to the psychological needs of adolescents and explore additional interventions (e.g., school-based CBT approach) (65) to prevent these issues from developing into more severe mental health problems.

### 4.2 Different trajectories on smartphone addiction

The relationship between smartphone addiction scores and different developmental trajectory groups is worth further exploring. The study found that although the initial levels of the Moderate-Increasing group were lower, their smartphone addiction scores were higher than those of the High-Decreasing group. This finding aligns with previous research, which indicates that individuals with continuously rising levels of depression and stress often lack effective coping strategies, and are more likely to rely on mobile phones as a tool for emotional regulation or reality escaping, leading to higher levels of smartphone addiction and other externalizing behaviors (50, 66, 67). This finding not only highlights the connection between smartphone addiction and negative emotions but also suggests that more proactive interventions are needed for groups with continuously increasing or relatively high levels of depression and stress. These interventions should help individuals develop healthier coping strategies and reduce their reliance on smartphones.

Moreover, the results of this study also reveal gender differences. Consistent with previous research, girls are more likely to perceive higher levels of stress and depression and are more likely to follow developmental trajectories where psychological problems gradually increase. In addition, this study found that girls have significantly higher scores on smartphone addiction than boys, but there is little change in the trend of mobile phone addiction across different stress and depression developmental trajectory groups, indicating that their stress and depression trajectories in different contexts are not strongly associated with mobile phone addiction. In other words, although girls may experience higher levels of stress or depression, their mobile phone addiction is not solely triggered by these psychological states. Previous studies suggest that this may be due to girls spending more time on social media and investing more emotional energy in their phones, thus leading to higher levels of addiction (68). This implies that intervention measures should not only target adolescents in high-risk trajectory groups but also focus on excessive dependence on smartphone usage among girls, for instance, educational programming on academic motivation and efficient time usage could be considered (65). Future research could further explore the reasons behind the higher levels of smartphone addiction in girls. Additionally, the absence of a significant interaction between gender and different trajectory group in relationship with smartphone addiction may be partly explained by the fact that females generally exhibited higher levels of smartphone addiction across all trajectory groups, rather than being concentrated within a specific subgroup. Another possible explanation is the lack of relevant moderating variables, such as reassurance seeking and fear of missing out, which might have obscured potential gender-related variations across different trajectory groups on smartphone addiction (69). Future research is recommended to include some moderators and apply multi-group latent growth modeling to further examine whether gender effects differ under varying conditions.

### 4.3 Limitations and future directions

While this study reveals the joint developmental trajectory and gender differences in perceived stress and depressive symptoms among adolescents, several limitations remain to be addressed. First, as the sample was drawn from a specific region and did not include clinical samples, the generalizability of the findings may be limited. Future research should consider selecting more diverse and heterogeneous samples to enhance the ecological validity and representative of the trajectory model. Second, in terms of data collection methods, the reliance on self-reported measures may introduce common method bias. Future studies could incorporate multiple measurement methods, such as teacher assessments, peer reports, or physiological indicators, to improve the reliability and validity of the findings. Third, concerning the tracking duration, this study only covers the psychological development window during middle school. However, the transition to high school, marked by increased academic pressure and growing autonomy, may reshape adolescents’ psychological adaptation trajectories (19). Extending the follow-up period to the critical developmental stage of 18 years would help to comprehensively map the dynamic psychological development during adolescence. Finally, while our study identifies the patterns in perceived stress and depressive symptoms trajectories among adolescents, the synergistic effects of internal and external factors remain underexplored. External systems (e.g., family dynamics, school climate, social networks) and individual characteristics (e.g., coping styles, emotional regulation) likely interact dynamically to shape psychological outcomes. Future research should prioritize multilevel analyses to examine how cultural contexts, gender norms, and digital behaviors collectively influence mental health trajectories across developmental stages.

## 5 Conclusion

Our findings reveal five distinct developmental trajectories of depressive symptoms and perceived stress among adolescents (Low-Stable, Moderate-Stable, Moderate-Increasing, High-Decreasing, High-Stable), and their impact on smartphone addiction. The study underscores the importance for schools and families to recognize both collective trends and individual differences in the multifaceted development of adolescent mental health. It highlights that timely intervention to alleviate negative emotions and stress perceptions is crucial, particularly for the more vulnerable girl population.

## Data Availability

The raw data supporting the conclusions of this article will be made available by the authors, without undue reservation.
